# Expanding the biodiversity of *Oenococcus oeni* through comparative genomics of apple cider and kombucha strains

**DOI:** 10.1186/s12864-019-5692-3

**Published:** 2019-05-02

**Authors:** Marc P. Lorentzen, Hugo Campbell-Sills, Tue S. Jorgensen, Tue K. Nielsen, Monika Coton, Emmanuel Coton, Lars Hansen, Patrick M. Lucas

**Affiliations:** 1 University of Bordeaux, ISVV, Unit Oenology, F-33882 Villenave d’Ornon, France; 2 Lallemand SAS, 19 Rue des Briquetiers, 31702 Blagnac, France; 3 Department of Environmental Science, Environmental Microbial Genomics Group, Aarhus University, Frederiksborgvej 399, 4000 Roskilde, Denmark; 4 Université de Brest, Laboratoire Universitaire de Biodiversité et Écologie Microbienne, EA 3882. ESIAB, Technopole Brest-Iroise, 29280 Plouzané, France

**Keywords:** *Oenococcus oeni*, Lactic acid bacteria, Comparative genomics, Phylogenomics, Pan-genome, Industrial microbiology

## Abstract

**Background:**

*Oenococcus oeni* is a lactic acid bacteria species adapted to the low pH, ethanol-rich environments of wine and cider fermentation, where it performs the crucial role of malolactic fermentation. It has a small genome and has lost the *mutS-mutL* DNA mismatch repair genes, making it a hypermutable and highly specialized species. Two main lineages of strains, named groups A and B, have been described to date, as well as other subgroups correlated to different types of wines or regions. A third group “C” has also been hypothesized based on sequence analysis, but it remains controversial. In this study we have elucidated the species population structure by sequencing 14 genomes of new strains isolated from cider and kombucha and performing comparative genomics analyses.

**Results:**

Sequence-based phylogenetic trees confirmed a population structure of 4 clades: The previously identified A and B, a third group “C” consisting of the new cider strains and a small subgroup of wine strains previously attributed to group B, and a fourth group “D” exclusively represented by kombucha strains. A pair of complete genomes from group C and D were compared to the circularized *O. oeni* PSU-1 strain reference genome and no genomic rearrangements were found. Phylogenetic trees, *K*-means clustering and pangenome gene clusters evidenced the existence of smaller, specialized subgroups of strains. Using the pangenome, genomic differences in stress resistance and biosynthetic pathways were found to uniquely distinguish the C and D clades.

**Conclusions:**

The obtained results, including the additional cider and kombucha strains, firmly established the *O. oeni* population structure. Group C does not appear as fully domesticated as group A to wine, but showed several unique patterns which may be due to ongoing specialization to the cider environment. Group D was shown to be the most divergent member of *O. oeni* to date, appearing as the closest to a pre-domestication state of the species.

**Electronic supplementary material:**

The online version of this article (10.1186/s12864-019-5692-3) contains supplementary material, which is available to authorized users.

## Background

*Oenococcus oeni* is the main lactic acid bacteria (LAB) species driving malolactic fermentation (MLF) in wine. The metabolic capabilities of *O. oeni* are of great interest due to its role in the wine industry, and by exploring its intraspecific biodiversity, we not only contribute to a better knowledge of the species and of potential domestication events, but also expand the toolbox of strain phenotypes that can be selected and used industrially [1, 2]. The species was first named “*Leuconostoc oenos*” on the basis of morphological and phenotypic similarities with the members of the *Leuconostoc* genus. However, it differs by its capacity to grow at low pH and is phylogenetically distant from other *Leuconostoc* species, which led to its reclassification in the *Oenococcus* genus in 1995 [3]. *O. oeni* is one of the three *Oenococcus* species described to date. The other two are *O. kitaharae*, isolated from distillation residues of Japanese Shochu [4] and *O. alcoholitolerans*, collected from Brazilian Cachaça and bioethanol plants [5].

*O. oeni* is rarely detected in the natural environment, even at the surface of grape berries in the vineyard [6]. In contrast, it is highly specialized to the wine environment thanks to its tolerance to low pH and high ethanol levels. Although it is a minor species in grape must, it develops faster than all other LABs during and after alcoholic fermentation and usually becomes the predominant bacterial species during MLF [7]. *O. oeni* was also frequently reported in French and Spanish apple cider where it also contributes to MLF [8, 9].

The first *O. oeni* genome sequence was released in 2005, from the strain PSU-1 [10]. This is a reference sequence not only because it was the first of this species, but also because it is the only complete genome reported to date, until this study. More recent studies have reported draft sequences of more than 200 strains originating from different wine types and regions [10–17]. Like many other LAB species, *O. oeni* has a rather small genome, ranging from 1.7 to 2.2 Mb, which most likely results from extensive loss of functions during specialization of the species to life in wine, a nutrient-rich environment [18]. The most striking feature of the *O. oeni* genome is that it lacks the *mutS-mutL* system involved in DNA mismatch repair. This makes *O. oeni* a “hypermutable” species that accumulates spontaneous mutations 100 to 1000 times faster than other LAB species [19]. The full genome of strain PSU-1 and genetic maps of 8 other strains showed that it contains only two sets of rRNA genes, whereas 4 to 9 are usually present in other LAB species [10, 20, 21]. The rRNA operon copy number probably correlates to the translational activity and growth kinetics of bacteria [22]. In agreement with this hypothesis, *O. oeni* is a fastidious and slow growing species compared to other LAB. The recent availability of numerous genome sequences has made it possible to analyze the genomic variations in this species. Recently a pangenome assembly demonstrated variations in sugar and amino acid metabolism and the distribution of competence genes [12, 14], and other studies have also reported genetic variations related to carbohydrate uptake and metabolism [23, 24], stress resistance [25, 26] and properties relevant to biotechnology [2, 27, 28].

Phylogenetic studies based on multilocus sequence typing (MLST) of numerous strains isolated from diverse sources have revealed that they fall within two major genetic groups, named A and B, with A strains found exclusively in wine, while B strains were found in both wine and cider [29–32]. A third group C containing only a single strain (IOEB_C52) isolated from cider was also hypothesized [13, 31]. Phylogenomic trees that were recently derived from genome sequences have confirmed the two phylogroups A and B, whereas a consensus had not yet been reached regarding the existence of the third group C [12, 13]. MLST and phylogenomics have also revealed subgroups of strains that correlate with different regions or product types such as cider, wine or champagne [13, 31]. Recently, strains from two different genetic subgroups were detected mainly in the Burgundy and Champagne regions [11, 33]. They preferentially develop in either red or white wine due to differences in their tolerance to low pH and phenolic compounds that differ between these two wine types [34].

The genomic specialization of *O. oeni* contrasts with other LAB species such as *L. plantarum*, the second most abundant LAB species in wine, whose genomic evolution appears to be detached from ecological constraints [35]. *L. plantarum* has a nomadic lifestyle, which allowed it to acquire many genetic functions, but not to specialize to any specific environment. It is present in many diverse environments, including wine, cider, kombucha or shochu [36–38]. However, although it grows faster than *O. oeni* in culture media, it does not outcompete *O. oeni* in the vast majority of wines.

The aim of this study was to clarify the population structure of *O. oeni* with the addition of new genomes from strains isolated from cider that were not assigned to either A or B groups [33] and strains isolated from kombucha, a fermented tea and an until recently unknown niche of *O. oeni* [38]. The 9 cider strains were selected on the basis of a genetic typing performed in a previous study which showed that they did not have the characteristics of either group A or B strains [33] and the 5 kombucha strains were selected on the basis of PCR-M13 profiles [38]. Complete or draft genomes of these strains were produced and analyzed along with all other *O. oeni* genomes reported to date in order to investigate their phylogenetic distribution and to identify genes involved in adaptation to their environment of isolation.

## Results

### De novo genome sequencing

To investigate *O. oeni* evolutionary history and to find markers of possible genomic adaptations to a different medium than wine, we sequenced the genomes of 14 strains that were recently isolated from cider (9 strains) and kombucha (5 strains) (Table 1). Two complete genomes - UBOCC-A-315001 (kombucha) and CRBO_1381 (cider) - and 12 draft genomes were produced with Illumina technology. Paired-End sequencing was used on all strains, and the two complete genomes were obtained with the addition of Mate-Pair reads to connect contigs and span the two repeat-filled ribosomal RNA regions of the genome. UBOCC-A-315001 was assembled into a single contig, while CRBO_1381’s six contigs were manually joined by bridging gaps with polymerase chain reactions (PCRs) to obtain the missing sequences. All genomes were annotated using MicroScope’s automatic annotation pipeline, and manual curation was carried out on the genome of UBOCC-A-315001 using the same pipeline [39, 40]. The superior, manual annotation was spread to all genes using a similarity criterion (> 90% identity, > 70% similarity, alignment > 80% of CDS length) to supersede the automatic annotation on a gene by gene basis.

**Table 1:** Newly sequenced genome assemblies and annotations. Kombucha strains were isolated from 3 separate fermentations by the same producer. (1) sequence reported in [10]

Strains	Assemblies				Annotation			Isolation		
	Length (bp)	Contigs	N50	L50	GC %	CDS	fCDS	Country	Type/Region	Year
UBOCC-A-315001	1,876,981	1	1,876,981	1	37.73	1858	47	France	Kombucha - Brittany - green tea ferment day 0 (biofilm)	2013
UBOCC-A-315002	1,821,972	160	29,861	15	38.05	1841	39	France	Kombucha - Brittany - black tea ferment starter (biofilm)	2014
UBOCC-A-315003	1,870,064	14	219,792	4	37.69	1923	21	France	Kombucha - Brittany - black tea ferment day 8 (liquid)	2014
UBOCC-A-315004	1,872,260	82	49,629	11	37.71	1904	83	France	Kombucha - Brittany - green tea ferment day 2 (liquid)	2014
UBOCC-A-315005	1,870,799	13	286,569	3	37.69	1917	18	France	Kombucha - Brittany - green tea ferment day 0 (liquid)	2013
CRBO_1381	1,834,577	1	1,834,577	1	37.81	1859	62	France	Cider - Normandy	1993
CRBO_1384	1,825,193	104	39,866	14	37.8	1917	41	France	Cider - Calvados	2008
CRBO_1386	1,788,970	43	124,72	6	37.79	1830	44	France	Cider - Normandy	1993
CRBO_1389	1,902,472	39	143,611	6	37.64	1932	70	France	Cider - Mayenne	2008
CRBO_1391	1,922,334	146	38,303	17	37.62	2004	46	France	Cider - Mayenne	2008
CRBO_1395	1,867,409	30	141,686	5	37.68	1902	34	France	Cider - Mayenne	2008
CRBO_13106	1,841,703	87	47,896	11	37.72	1910	35	Spain	Cider - Asturias	2006
CRBO_13108	1,885,467	41	126,048	5	37.7	1936	57	France	Cider - Normandy	2008
CRBO_13120	1,860,062	182	19,393	25	37.78	1981	74	France	Cider - Calvados	2008
PSU-1^(1)^	1,780,517	1	1,780,517	1	37.89	1859	159	–	–	–

The newly sequenced genomes range from 1.79 to 1.92 Mb in size, which is in the range of *O. oeni* genomes reported to date (from 1.69 to 2.55 Mb according to data in Genbank). The two full genomes contain only two sets of rRNA operons, which seems to be universal in this species. The count of coding regions (CDS) is fairly stable through the assemblies at a mean of 1905 ± 48, though high numbers of contigs in several assemblies may inflate the CDS count when genes are counted more than once. The complete genomes converge at 1859 CDSs, though with a drastic difference in pseudogenes (fCDS); PSU-1 carries more pseudogenes than any of the other assemblies.

### Phylogenetic clustering of the newly sequenced strains

**Fig. 1:**
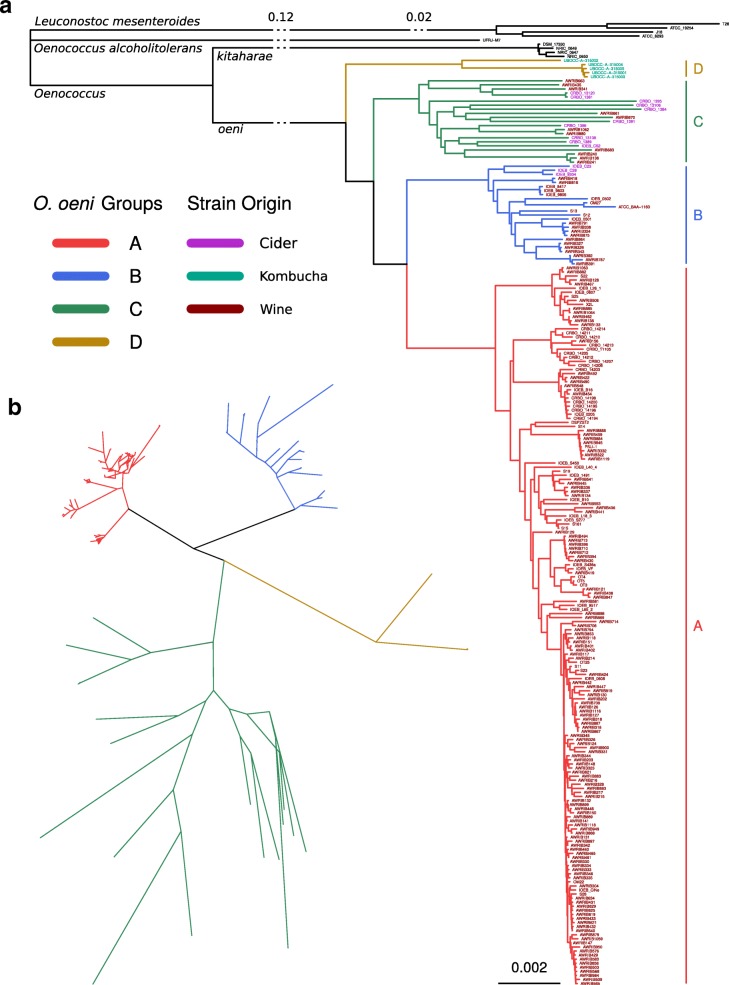
Phylogenetic clustering of all studied *O. oeni* strains. **a** Neighbor Joining hybrid phylogenetic tree based on distance matrices calculated with Average Nucleotide Identity MUMmer and BLAST. **b** Maximum Likelihood trees based on the identified 210,180 SNPs from the *O. oeni* coregenome

To identify the phylogeny of the newly sequenced strains, phylogenetic trees were constructed using the 14 obtained genome sequences as well as 212 *O. oeni* genome assemblies from NCBI’s Genbank. Genome sequences of *O. kitaharae*, *O. alcoholitolerans* and *Leuconostoc mesenteroides* were used as outgroups. A phylogenetic tree was constructed using the Average Nucleotide Identity (ANI) method, using a combination of BLAST and MUMmer to find the optimal distances inside and between the species, respectively. ANIm and ANIb distance matrices were used to reconstruct a hybrid tree by using Neighbor Joining (Fig. 1a)*.* The previously identified A and B groups were well separated in this tree and subgroups are clearly visible in A as reported in previous studies [12, 13]. Group A may also be oversampled, judging from the little if any evolutionary distance between numerous strains located at the extremity of the tree. The 9 additional cider strains analyzed in this work were all grouped into a single clade, along with 11 strains isolated from Australian wines that were previously labelled as group B, but no other wine strains. The strain IOEB_C52, which was isolated from cider and previously attributed to the hypothetic group C [13, 31] was also placed in this clade. Consequently, we continued the nomenclature and named the clade group C. The 5 kombucha strains were the most dissimilar to the rest of the studied *O. oeni* strains. They clustered in a separate clade, which we termed group D. However, this group had two branches, one of which consisted of 4 almost identical strains – suggesting that the biodiversity of the newly discovered clade was not represented well with current genomes. Indeed, the similarity of 4 of the genomes indicated that the corresponding isolates might belong to the same strain. Interestingly, the two 2013 isolates were obtained from different kombucha fermentations than the 2014 ones. The fact that the same strains were detected in the fermentation despite the different batch and tea type suggested that it remained present in the production environment 1 year later. It was striking that the evolutionary distances inside the C, and to some degree D, group were much larger than those in group A, when comparing the branch length to the clades’ earliest shared node. Two possible options could explain this observation: The C clade may have been under-sampled, or there could be a higher rate of mutation of these strains compared to the other groups. It may be that group C strains are present at the start of fermentation, but is selected against during the fermentation and thus not present at the end, where most strains are isolated. This survivor bias could be a cause of undersampling, whereas the less restrictive cider environment allows a higher diversity of strains to grow. It has been suggested that *O. oeni* strains are not generally constrained by geography [33], so we did not consider that the divergence was due to the fact that these strains evolved independently due to geographical partitioning.

To confirm the existence of the two newly defined groups C and D by another analytical method, we calculated distance matrices from the presence of Single Nucleotide Polymorphisms (SNPs). The core genome of all new and public *O. oeni* strains (*n* = 226) was calculated and aligned by ClustalOmega. 210,180 SNPs were identified and used to reconstruct phylogenetic trees using Maximum Parsimony (data not shown) and Maximum Likelihood showing evolutionary distances (Fig. 1b). Both trees confirmed the distribution of strains into the same four clades as described above. Evolutionary distances revealed by Maximum Likelihood also confirmed the much larger evolutionary distances in group C compared to those observed in the A or B groups (Fig. 1b).

Domestication of wine-specialized strains of *Saccharomyces cerevisiae* has been estimated to have occurred around 9200 years ago [41], but the domestication of *O. oeni* from a low ethanol environment niche (rotting fruits in nature) to industrial wine production has not yet been well described, and it remains to be determined when *O. oeni* gained its current role in MLF. Group A strains are by far the most commonly isolated strains in wine, containing virtually all commercial strains, and therefore appear to be the best adapted to the ecological niche [12, 13, 34]. Conversely, group C strains have been isolated the most from cider, and group D strains have only been isolated from kombucha. The structure of the phylogenetic tree (Fig. 1a) showed the clear divergence of the sub-populations of *O. oeni*. The tree lacks strain isolation dates, but most have roughly the same total branch lengths, which would indicate equal rates of genetic evolution. Group C strains displayed a greater intra-clade distance than A or B (Fig. 1b), which might indicate that the group contains subpopulations adapting to more diverse environments and possibly meriting a future subdivision of the clade. A more comprehensive overview of the *O. oeni* population in non-wine environments would likely shed light on this issue to more clearly define the specialized niche of each phylogenetic group.

### Synteny and variable regions in full genomes of C and D group strains

To determine if C and D group strains shared the same genome organization as that of group A strains, we circularized the genomes of one representative strain from each group: CRBO_1381 (group C) and UBOCC-A-315001 (group D). They are the first fully completed *O. oeni* genomes since PSU-1 (group A), although another full genome has been uploaded to the NCBI’s database during the preparation of this manuscript (strain “19”, GCA_003264795.1). The new genomes are 1,834,577 and 1,876,981-bp long, respectively, and contain two sets of rRNA operons, which is somewhat similar to PSU-1’s genome (Table 1). Genomic rearrangements amongst group A, C and D strains were investigated using the SyMap algorithm [42], but no rearrangements or inversions were found (Additional file.1: Figure S1).

Although they are closely related, strains in the C and D groups hold specific genetic regions that were identified by comparing the two complete genomes against all the genomes of the other group (Additional file.2: Figure S2, Additional file.8: Table S1). The UBOCC-A-315001 strain counts 6 variable regions for a total of 208,765 bp and 273 CDS which are not present in the 21 group C genomes, while the CRBO_1381 strain has 10 variable regions, 143,095 bp and 177 CDS, not detected in the 5 group D strains.

### Pangenome analysis

**Fig. 2:**
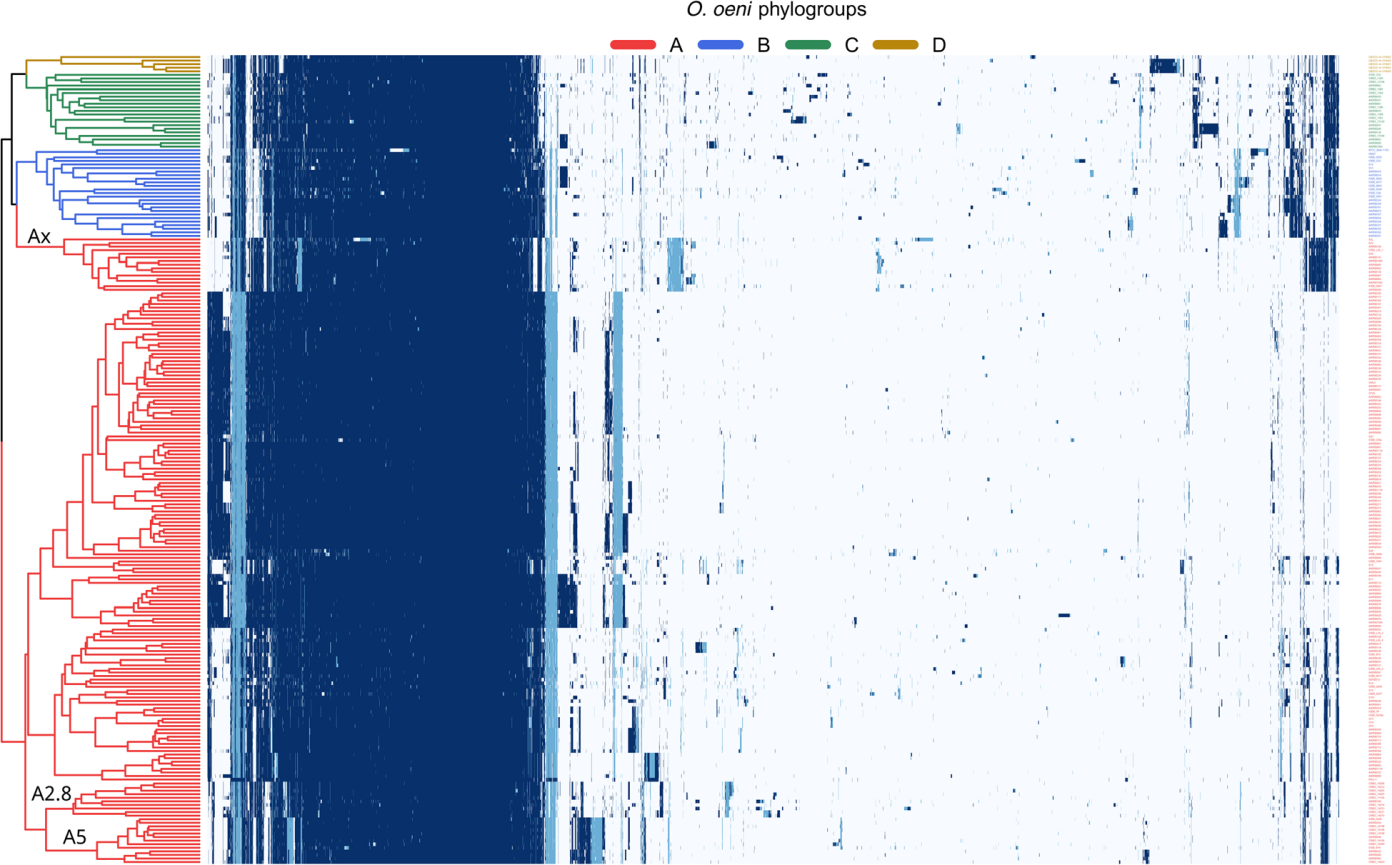
Heatmap of the *O. oeni* pangenome. 226 strains are represented on the rows and clustered by complete linkage. Genes are binned into families (MICFAMs) with threshold parameters set to > 80% amino acid identity and > 80% alignment coverage, making 6051 columns (clustered with complete linkage). Genes annotated as ‘fragment’ are displayed in light blue, and MICFAMs with only 1 entry were excluded (3385 singletons). Strains comprised in subgroups A5 and A2.8 are indicated in the dendrogram according to Campbell-Sills et al. (2017). Subgroup Ax was delineated from this figure

Previous work has defined a pan genome assembly of Oenococcus based on 191 strains [12]. In order to more robustly identify unique genetic properties of strains of group C and D, a pangenome was calculated and analyzed for the 226 O. oeni strains. MicroScope’s pangenome utility was used to count gene families (MICFAMs) using threshold parameters set to > 80% amino acid identity and > 80% alignment coverage. This resulted in a total of 9436 unique MICFAMs (the pangenome), of which 892 MICFAMs were present in all strains (the coregenome). The size of the core genome approached a plateau, while the progression of the pangenome did not level off (Additional file.3: Figure S3). Group A exhibited the highest amount of MICFAMs in the variable genome and slightly more total MICFAMs than groups C and D (Table 2), though this may partially be due to higher numbers of fragmented genes and the higher volume of sequenced strains of group A. A heatmap of all MICFAMs in all genomes was constructed to visualize their distribution (Fig. 2). Both axes of the heatmap were clustered by complete linkage, and the resulting dendrogram was displayed for the strains. The population structure in the dendrogram was similar to that of the phylogenetic tree of Fig. 1, dividing all the strains into the same four A, B, C and D groups, thus demonstrating that each clade has specific gene content. The heatmap clearly showed that each group of strains differs from other groups by the presence or absence of a number of MICFAMs. Several subgroups of strains were also discernible according to the heatmap and the dendrogram. For example, we observed the clustering of the recently described A5 and A2.8 subgroups that are predominantly made up of strains adapted to red and white wines, respectively [11, 34]. Interestingly, one A subgroup, that we named Ax, was found to be an outlier, being clustered closer to group B. This subgroup showed a unique genetic pattern, indicating that specific adaptation may have occurred.

**Table 2:** MICFAM distribution of the variable genome. Strains were randomly sampled for MICFAMs and singletons, reported either with duplicate entries removed (unique) or with the total number

Variable genome				Bootstrap (*n* = 5; 10,000 reps)			
Group	Strains	Unique MICFAMs	Unique Singletons	Unique MICFAMs mean ± SD	Unique Singletons mean ± SD	MICFAMs mean ± SD	Singletons mean ± SD
A	175	3843	2002	1607 ± 139	57 ± 53	5458 ± 140	57 ± 52
B	25	2356	509	1512 ± 99	106 ± 65	5345 ± 67	107 ± 65
C	21	2251	561	1513 ± 93	141 ± 57	5153 ± 100	141 ± 57
D	5	1049	41	1043 ± 12	69 ± 37	5094 ± 13	68 ± 37

### Genes associated with environmental specialization

**Fig. 3:**
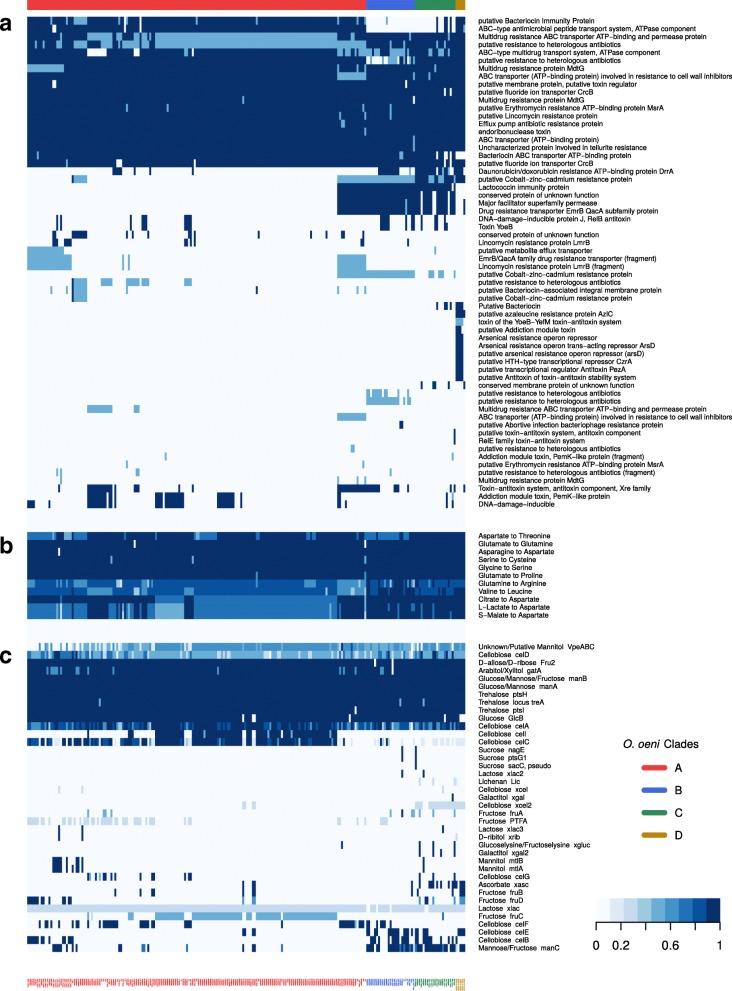
Heatmaps of pangenome categories. **a** Distribution of genes amongst 226 *O. oeni* strains annotated for “resistance” or “toxin”, **b** amino acid biosynthesis pathways or **c** Phosphotransferase systems. Orientation of the heatmaps is transposed from Fig. 2, listing strains on the columns and MICFAMs on the rows. The order of the strains is the same as in the pangenome, while the MICFAMs were clustered anew (complete linkage). Phylogenetic groups A-D and strain names are indicated on the top and bottom, respectively. In **a**, light blue color indicates the presence of a gene fragment. In **b**, pathway completion is colored according to KEGG metabolic pathway maps. (Note that some pathways overlap). In **c**, light to dark colors indicate the number of components present for a given PTS (between 1 and 4), listed by substrate and gene/transporter name

Using the pangenome, it was possible to search for genes (or their absence) that help explain the specialization of groups C and D strains to their environment. As several genes in the unique C and D clusters indicated a difference in stress or antibiotic resistance genes, we produced a slice of the pangenome listing only genes annotated with ‘Resistance’ or ‘Toxin’ terms (Fig. 3a). It was immediately apparent that members of the B, C and D groups possessed a block of genes not found in A, with the exception of the outlier subgroup Ax. This block of genes included a toxin/antitoxin component, a drug resistance transporter, a permease of the major facilitator family, a lactococcin immunity protein and a toxin ATP-binding protein, plus several other proteins only present in a few strains per group. This indicated that the strains of Group C and D, as well as B, retained or had gained more genes possibly related to survival outside of the high alcohol/low pH wine environment, which the more specialized group A strains had lost.

Group D strains differed from those of group B and most of C by the presence of a bacteriocin immunity protein, a putative antimicrobial peptide transporter, a putative azaleucine resistance protein and a cobalt-zinc-cadmium resistance protein. Several other proteins involved in various resistances and in the production of toxins or bacteriocins were also detected almost exclusively in group D (Fig. 3a). In addition, investigation by genome browser found a region coding for an arsenical operon present in 1 of the 2 group D strain branches. Interestingly, this region also contained a 4-gene operon for producing streptolysin S, which was found to be syntenic with several *Clostridium* and *Streptococcus* species (*sagB-D* genes and a small gene of unknown function) (Additional file.4: Figure S4). Two gene fragments were found in the vicinity of the streptolysin genes that hint at the possible gene transfer event: a putative conjugation nicking enzyme gene and a transposon gamma-delta resolvase. Comparison to *Streptococcus pyogenes*, which expresses the toxin [43], showed that at least two genes were missing in the operon, including the self-immunity protein *sagE* [44].

**Fig. 4:**
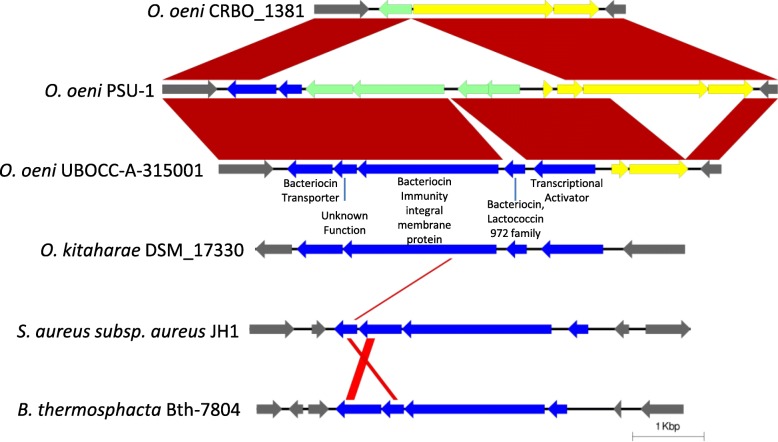
Comparison of genomic regions overlapping a bacteriocin operon. Pairwise BLAST hits shown in red (*e* < 0.001). Blue: Bacteriocin-related genes. Pale green: pseudogenes. Yellow: Genes of unknown significance of pangenome categories. Grey: Genes outside syntenic operon. Related genes detected by synteny at minimum 20% protein identity

Genome browser investigations also revealed that bacteriocin genes are grouped in a 5 gene operon (Fig. 4). This bacteriocin operon (putatively belonging to the lactococcin 972 family) encoded a transcriptional regulator, the bacteriocin-producing gene, an immunity protein, a transporter and a gene of unknown function. Only group D strains, with the exception of UBOCC-A-315002, possessed the bacteriocin-producing gene. The immunity gene was missing from the groups B, C and part of A. These groups did have a separate lactococcin immunity gene elsewhere in the genome, albeit in a region with numerous pseudogenes and without transcriptional regulators. Interestingly, the complete operon, including the lactococcin immunity protein, was also present in the outlier subgroup Ax and in 4 C strains, which were the only genomes to possess both versions of the immunity proteins.

Sternes [12] showed deficiencies in amino acid biosynthesis pathways, especially of group A strains. To further evaluate the adaptation of group C and D strains, we analyzed the distribution of amino acid biosynthetic pathways (Fig. 3b) and of phosphotransferase systems (PTSs) for sugar (Fig. 3b). It was apparent that most group C and D strains had the full complement of genes of the aspartate biosynthesis pathway, which many group A strains had lacked. The valine to leucine pathway provided more evidence to distinguish the groups: B and D were mostly competent, while C and A were almost entirely deficient. The aspartate to threonine pathway, on the other hand, was present in both C and D group strains, but missing in B strains, thus showing diversity despite that both B and C isolates were from cider.

PTSs were identified by searching through the MICFAM annotation. However, annotation of PTS is difficult due to their high similarity and because a given PTS can have multiple sugars as substrates. For this reason, we used the Transporter Classification Database to confirm the specificities of the MICFAMs [45], as well as the previously described *O. oeni* PTS proteins [24]. Five PTSs were complete in almost all strains, which could be considered as the basic set of PTSs (Fig. 3c). This set of PTSs was contrasted by the previous pangenome analysis [12], in which four main PTSs were found. The difference was likely due to incorporation of curated information on *O. oeni* PTS protein specificity [24]. Group C and D strains were delineated from group A, along with group B, in the distribution of cellobiose-specific PTSs, where the *celB* and *celE* variants were predominantly found. An ascorbate-specific system was previously described by Sternes [12] in strains that were attributed to group B, but that actually belong to group C in our analysis. It was also found in group D, although not in every strain of either group.

Furthermore, there were several versions of a cellobiose-PTS distributed throughout the population, although many strains had a few components of two or three different versions, but no ‘full’ PTS. This could be due to errors in assigning the MICFAMs, due to high similarity, or simply because the components of the different systems were able to fit together to form a functional PTS. The same might apply to the systems in which only one component was found, though misannotation or gene fragmentation also seemed likely. This was likely the same case for *fruB*, for which a version was almost uniquely shared between D and very few B and A strains, and for *fruD*, which appears as ‘fragments’ in the strains that also carry *fruB*, probably as a false positive. The different versions of the fructose PTS system were significant, because they enable the homofermentative metabolism of the sugar when it enters the cell as Fructose-1-phosphate, while the other transporters that could import fructose all lead to the phosphogluconate pathway [23].

One of the main methods in bacteria for acquiring new genetic information is transformation, where exogenous DNA is transported across the cell membrane and re-established as plasmids or in-cooperated into the bacterial genome [46]. However, pangenomic analysis of *O. oeni* has revealed that several of the genes involved in natural competence has suffered frameshift mutations, especially in group A strains [12]. We confirmed that the potential gene decay was more pronounced in group A strains, which showed showed gene fragmentation or absence of *comEA*, *comFC*, *comGA*, *comGC* and to a smaller extent *comGD*, which supported the hypothesis that this group had specialized the most to the wine environment and thus no longer required or benefited from the natural competence machinery [12]. Group C and D strains exhibited similar gene patterns as group B strains, possessing mostly intact *comC*, *comEA*, *comEC*, *comFA*, *comGA*, *comGC*, *comGF* and *comX* genes (Additional file.5: Figure S5). In addition, *comFC*, *comGB* and *comGD* were fully present in group C and D strains, whereas some B strains had suffered mutations. In addition, *comFC*, *comGB* and *comGD* were fully present in group C and D strains, whereas some B strains had suffered mutations. In addition, the *comC* gene was identified in just two group C strains. Thus, these findings showed that the two newly defined groups were the closest to having a full set of genes for natural competence, which was likely present in the ancestral progenitor of *O. oeni*. It remains to be experimentally verified if the state of these genes are sufficient to allow group C or D strains to be transformed.

Finally, given the absolute importance of the malolactic pathway for the MLF, we examined the presence and integrity of the three genes of this pathway in the newly sequenced strains and found for 1 of 2 group D strain branches a stop mutation in the *mleR* gene that encoded the positive transcriptional regulator MleR [47] (Additional file.6: Figure S6). Due to the adaptation of *O. oeni* to the wine environment, where the malolactic reaction likely helps the survival of the bacterium [48], the loss of regulation indicated a possible insensitivity to malic acid. The loss therefore dovetailed with the fact that the D strains were isolated from an environment known to contain only low levels of malic acid.

## Discussion

Genome analysis of *O. oeni* strains isolated from wine, cider and kombucha allows to better understand the evolution and adaptation of this species to its environments of origin. Wine is an inhospitable environment, mainly due to low pH (3.0–4.0) and high ethanol percentage (9–16%). *O. oeni* has adapted to this niche by developing a greater tolerance to the associated stresses – especially pH – than other LAB [35]. Fermented cider presents an environment similar to that of wine with regards to stress factors and available substrates. The pH level in cider is slightly higher (3.3–4.2), but the ethanol content is lower than wine (1.5–8%) [9, 49, 50]. Kombucha is made by fermenting sweetened tea with a symbiotic consortium of bacteria and yeasts [51]. The pH drops close to 3.0 during fermentation, but contains only trace levels of ethanol (0–1%).

We found that the 9 newly sequenced cider strain genomes clearly formed a clade of their own, joined with 11 wine strain genomes previously assigned to group B [12]. Given that *O. oeni* is well disseminated in the environment [33], the isolation of group C strains from cider and only a small number of Australian wines led us to believe that the group is not as well adapted to wine. The fact that some of the strains were isolated in wine does not invalidate this theory, because wine is investigated much more frequently than cider, meaning that cider-specialized strains that were present as a minor population might have been sampled. Group B also contains a small group of strains isolated from cider [13]. In both cases, more genomes and more samples from cider and other specific environments are of great interest to elucidate the specificity of *O. oeni* populations. The same issue applies to the group D strains. It is unknown if it is the only group of *O. oeni* strains that develops in kombucha, or if group D develops in other fermenting environments.

The synteny analysis of the three fully circularized genomes revealed no major genomic rearrangements. However, pangenome analysis revealed group and subgroup-specific gene clusters that generally support the phylogenetic trees and the delineation of specialized subgroups. The structure of subgroups was also supported by unsupervised clustering.

It is a normal process for species to lose biosynthetic pathways during the domestication process, and to instead acquire transporters for the required metabolites in their environment [52]. Members of group A have, by far, lost the most genes related to amino acid synthesis, demonstrating a greater degree of domestication than the others [53], where deficiencies in especially leucine and arginine — but also threonine and aspartate — biosynthesis have been identified [12]. As a result, several biosynthetic pathways are incomplete. Group D strains have suffered the smallest loss of amino acid biosynthetic capacity. Interestingly, subsets of group B and C strains both show deficiencies in arginine and leucine production pathways (though group C strains are less complete on average), while group C does not share the loss of the threonine pathway. Wine is a rich medium and before the onset of MLF, yeast autolysis make nutrients available for the propagation of the *O. oeni* population, though the release of threonine is less abundant than the amino acids of the other affected pathways [54]. Thus the gene loss is well explained by the availability of amino acids in wine. Since the group D strains has suffered less gene decay, its environment is probably less rich in free amino acids. The lack of uniform distributions of pathway completion may indicate an ongoing selection that is not equally advanced in all subgroups.

The niche of *O. oeni* is inhospitable to most bacteria and as such decreases the importance for antibiotic production or resistance genes. In two studies of 145 and 155 LAB isolates from MLF, only 10 and 5% of the strains produced bacteriocins, and none were from *O. oeni* [55, 56]. Group D strains alone possessed what appeared to be a full bacteriocin operon that matched the operon found in *O. kitaharae* [57], although the bacteriocin-producing gene was missing in one of the two strains. The lack of a functional malolactic operon in one of the D group strains is another point of similarity to *O. kitaharae*, which decreases tolerance to environmental stress [58, 59]. If the production of bacteriocin by these strains can be experimentally validated, it will underline the difference in environment, as these strains require tools to compete directly with other bacteria. The group C strains, on the other hand, displayed no drastic difference in toxin production or resistance genes compared to group B.

The pattern of fragmentation of certain genes may be an example of the process of adaptation. The “putative resistance to heterologous antibiotics” gene in Fig. 3 is actually a pair of adjacent, identically named genes of ~ 1500 and ~ 500 bp and was shown to contribute to resistance to antimicrobial compounds in *Bacillus subtilis* [60]. However, both genes only remain intact in a minority of strains. Group D and most of group C retain the whole genes, whereas either one is fragmented in virtually all of A and B. Curiously, almost no strains have suffered fragmentation in both at once. This suggests that either one contributes to survival. The surrounding genetic region is completely syntenic between strains of all groups, indicating its presence in a common ancestor. The pair of genes only remain complete in group D and parts of group C, and everywhere else they are decaying due to selection pressure in an environment where the full set is unnecessary for survival.

As mentioned previously, the D strains are split into two branches, with one outlier strain vs the rest (*n* = 4). There is a big inserted sequence in D which contains several resistance genes, but this insertion does not account for the branch split, as branch lengths are similar when calculated purely from the core genome. Even discounting the insert, the D strains are enriched with resistance genes not found in the rest of *O. oeni*. This can be explained by a potential need for more competitive abilities, since the D strains cannot depend upon the environment to prevent growth of other bacteria as much as the wine-strains can. The actual activity of the clade-specific gene clusters, including the bacteriocin-operon, arsenical resistance operon, cobalt-zinc-cadmium gene, and streptolysin operon, should be further investigated and validated experimentally.

## Conclusions

In this study, we expanded the knowledge of the *O. oeni* population structure using new genome sequences from cider and kombucha. This led to the integration of two additional phylogenetic groups. Here, we provide evidence to chart their evolutionary history using sequence-based methods and gene absence/presence patterns. The pangenome represents a powerful tool for analyzing strains through a genome browser by synteny to other strains, and by gene classifications like COGs [61]. This makes it simple to search for strains with specific characteristics. In the future, addition of new, complete *O. oeni* genomes can easily be compared to the public database to find specific adaptative traits. Several gene clusters in the pangenome subgroups remain to be identified or linked to an actual phenotype. Protein characterizations and better computational tools may lead to improvements in annotation, which is required to better understand how the strain genotype influences its phenotype. The presence of these gene clusters should make it possible to identify the genes driving adaptation to specific environments.

## Methods

### Genome sequencing

Strains were isolated from French cider and kombucha and grown in grape juice medium (per 1 L: 250 ml grape juice, 5 g yeast extract, 1 ml Tween 80, adjusted to pH 4.8). DNA isolation was performed with a standard Wizard Genomic DNA Purification kit (Promega, WI, USA), for which the protocol was modified with the addition of 1 h of lysozyme treatment and longer centrifuge times to optimize yield (up to 30 min). The purity of the extracted DNA was tested by Biospec-nano, (Shimadzu Biotech, Japan) and quantified on a microplate fluorescence reader (SpectraMax M2, Molecular Devices, CA, USA) using iQuant (HS kit, GeneCopoeia, MD, USA) or Qubit (Thermofisher, MA, USA).

DNA libraries were prepared with Illumina Nextera Paired-End or Mate-Pair protocols (Illumina, CA, USA). 1/4 input DNA was used for the Mate-Pair gel-plus protocol on a BluePippin machine (Sage Science, Beverly, MA, USA). 6–8 Kb and 8–10 Kb fractions were selected using a pulse field program with a 0.75% cassette. A Covaris E220 machine was used to fragment the DNA prior to Mate-Pair sequencing library construction with the following parameters: target: 500 nt, intensity: 3, duty cycle: 5%, cycles/burst: 200, treatment time: 80s.

The libraries were sequenced on an Illumina Miseq with 2 × 250 bp reads. Reads were cleaned with Cutadapt 1.12 [62], evaluated with fastQC 0.11.5 [63] and four different assemblers (SPAdes 3.6.2 [64], Minia 3 [65], Velvet 1.2.10 [66], MIRA 4.9.5_2 [67]) that were each tested with different parameters to find the best assemblies, evaluated by the N50 metric. SPAdes with the ‘careful’ option enabled was chosen to assemble the genomes, and QUAST [68] was used to calculate genome assembly statistics. Assembly accession numbers are given in Additional file.9: Table S2.

### PCR bridging

To circularize CRBO_1381, the assembly scaffold was used to identify regions of ‘N’s and Primer3 0.4.0 [69] was used to make primers to bridge these ‘N’ gaps, with default primer design settings and with a target size of 1 kb or less, essentially placing the primer as close to the end of the known sequence as possible to obtain as much new information as possible with dye-terminator sequencing. Primer sequences and targets are provided in Additional file.10: Table S3. PCR was performed with standard settings using standard *Taq* DNA polymerase (New England Biolabs, Ipswich, MA, USA), product size was determined by agarose gel or multiNA, concentration by fluorescence (iQuant) or multiNA (Shimadzu, Japan), and sequencing was performed by Eurofins Genomics (Ebersberg, Germany).

### Public genomes

*O. oeni* genomes (*n* = 213) was found on NCBI’s Genbank. Among these, 142 were reported, but uploaded only as raw reads instead of assembled genomes [12]. In order to use them in the analysis, we downloaded the sequencing data from NCBI and assembled them, using the same procedure as with our own reads. Of the resulting genomes, 1 was discarded, 130 were assembled by SPAdes 3.6.2 and 11 by MIRA 4.9.5_2, resulting in a total of 212 public genomes (provided in Additional file.11: Table S4), along with the non-*oeni* genomes).

### Genome annotation

The newly sequenced genomes were annotated using the automatic pipeline of LABGeM’s MicroScope service [70]. Before submission to the annotation service, all Ns and degenerate bases were purged from the genomic sequences to satisfy MicroScope requirements, though this was only relevant for very few genomes. Several algorithms and databases were used for annotation, both for the automatic pipeline and manual curation: Prodigal, Glimmer and AMIgene algorithms for gene detection. SwissProt, TrEMBL protein databases for gene identification. PRIAM EC, MetaCyc Pathways, COGnitor, EGGNOG and FigFam databases for predicting function. For each gene, the pipeline attempts to identify genes from a set of rules, using BLAST to find similarity in described sequences in the databases. If computational evidence exists (e.g. similarity in PRIAM EC or FigFam), but no sequence exists in the protein databases, the gene identity is labeled ‘putative’.

Manual annotation was done by inspecting the combined results from protein databases, functional predictions and synteny information. The combination of sources allowed the curator to infer gene identities and functions in cases where the automatic annotation could not.

In order to use the MicroScope genome browser (MaGe) and compare the new genomes to previously assembled sequences, we submitted the 14 new genomes, as well as the public genomes, to the annotation pipeline [40].

### Phylogenetic trees

ANI is a measure that aligns a genome to all other genomes to determine evolutionary distance [71]. To root the tree, related *Oenococcus* species were included, namely *O. kitaharae* and *O. alcoholitolerans*, as well as the closest non-*Oenococcus Leuconostocaceae*, *Leuconostoc mesenteroides*. The tree was clustered by Neighbor Joining and rooted on *L. mesenteroides* (Fig. 1a). The ANI distance matrix was calculated with pyani 0.2.7 [72]. Both BLAST (ANIb) and MUMmer (ANIm) were used to circumvent their respective weaknesses, ANIm being better at calculating distances of closely related genomes, while ANIb is better at calculating distances between organisms of different species [73]. ANIb breaks up the sequences in small fragments for alignment, while ANIm does not. A hybrid distance matrix was produced to most accurately show the results, using ANIm for intra-species distances and ANIb for inter-species distances.

To obtain SNP data, the pangenome of *O. oeni* was calculated by MicroScope’s Pangenome tool [40] and 892 gene families were found. Among these, 723 contained no fragmented sequences. They were aligned with a custom script and Clustal Omega [74]. SNPs and indels (*n* = 218,180) were identified (excluding ‘N’s) and concatenated with another custom script. Both scripts were written in python 2.7 [75] using Biopython [76] and are available in the repository: https://github.com/marcgall/Genomics-01.

Initially, an unrooted phylogenetic tree was constructed using Neighbor Joining and the tree structure was confirmed by bootstrapping (*n* = 100) (Additional file.7: Figure S7). To confirm the structure with more robust methods, an unrooted phylogenetic tree was constructed using Maximum Parsimony (which computes distances by minimizing the number of changes) (data not shown1). Maximum Parsimony shows the structure of the phylogeny, but without the proper distances between clades. For this reason, a Maximum Likelihood tree was also constructed and plotted by Neighbor Joining to better show evolutionary distances (Fig. 1b).

All phylogenetic calculations (except for ANI) and plotting were done in R 3.4.4 [77] with RStudio1.0.143 [78], using dplyr 0.7.6 [79] and several Bioconductor packages to handle data [80]. Biostrings 2.46.0 was used to import sequences into R [81], APE 5.1 was used for Neighbor-Joining and bootstrap [82], phangorn 2.4.0 was used for Maximum Parsimony and Likelihood [83], dendextend 1.8.0 for dendrogram handling [84] and ggtree 1.10.5 for plotting trees [85].

### Pangenome

The pangenome was calculated by the Pangenome tool in MicroScope [40]. The core and variable genome files were combined to make a matrix showing presence/fragmentation/absence of every MICFAM in R [77], discounting all singletons because they are not assigned a MICFAM ID by the Pangenome tool. The rows and columns of the matrix were clustered using hclust with complete linkage and plotted as a heatmap using gplots 3.0.1 [86] and RColorBrewer 1.1–2 [87] for coloring. Dendextend was used for dendrogram handling [84].

Genome accession and gene loci for bacteriocin and streptolysin S synteny comparisons are provided in Additional file.12: Table S5.

PTS genes were identified as described in Results, but not all gene names were provided. In these cases, a placeholder gene name was added with the putative substrate name, e.g. ‘xlac1’ for a lactose PTS.

## Additional files

Additional file 1: **Figure S1.** Whole Genome Synteny Dotplot. Sequences of CRBO_1381 and UBOCC-A-315001 were compared against PSU-1 using SyMap. The algorithm finds pairwise genome alignment ‘anchors’ - represented by dots - and computes blocks of synteny (PDF 11 kb)

Additional file 2: **Figure S2.** Variable regions in groups C and D genomes. MicroScope RGP-finder was used to identify specific regions of (**a**) group C strain CRBO_1381 against the 5 group D strains and of (**b**) group D strain UBOCC-A-315001 compared to the 21 group C strains. Specific regions are shown in grey. Supporting algorithms are shown in blue and black (Interpolated Variable Order Motifs and Regions of Genomic Plasticity). tRNAs are in pink. (**c**) MaGe’s RGP-finder tool was employed to locate all variable regions, determine their size and the number of CDS they contain (PDF 466 kb)

Additional file 3: **Figure S3.** Size of the pangenome at any given number of strains. At every step, 10 combinations of strains were randomly sampled within the total distribution. A locally weighting smoothing (loess) regression line was drawn for both sets (PDF 232 kb)

Additional file 4: **Figure S4.** Comparison of genomic regions overlapping a streptolysin operon. Pairwise BLAST hits shown in red (*e* < 0.001), darker color indicates better alignment. Blue: Streptolysin-associated genes. Grey: Genes outside syntenic operon. Related genes detected by synteny at minimum 26% protein identity (PDF 62 kb)

Additional file 5: **Figure S5.** Competence genes identified in the pangenome. Gene presence in blue, fragments in light blue (PDF 22 kb)

Additional file 6: **Figure S6.** Schematic representation of the stop mutation disrupting the malolactic transcriptional regulator in group D strains compared with PSU1 (PDF 29 kb)

Additional file 7: **Figure S7.** Fortified Neighbor Joining phylogram. Calculated from core SNP data with Kimura 2-parameter distances, bootstrap *n* = 100 (PDF 717 kb)

Additional file 8: **Table S1.** Variable regions in groups C and D genomes, gene overview (XLSX 29 kb)

Additional file 9: **Table S2.** Newly sequenced genome assembly accession numbers (XLSX 5 kb)

Additional file 10: **Table S3.** Primer list. The sequence surrounding NNN-islands in the CRBO_1381 assembly scaffold was entered into Primer3 with default settings (GC clamp = 1) to find suitable primersets for PCR product sequencing. The target product size, discounting Ns, was 1 kb. Primersets were tested with Primer-BLAST on PSU-1. PCR product size was tested by agarose gel and multiNA and sequenced by Eurofins Genomics (XLSX 5 kb)

Additional file 11: **Table S4** Public genome accession numbers (XLSX 12 kb)

Additional file 12: **Table S5** Genomes and gene loci used for bacteriocin- and streptolysin S synteny comparison (XLSX 5 kb)

## Abbreviations

ANI: Average nucleotide identity
ANIh: ANI hybrid
ANIm: ANI MUMmer
CDS: Coding region
LAB: Lactic acid bacteria
MICFAM: (Pangenome) gene family
MLF: Malolactic fermentation
MLST: Multilocus sequence typing
PCR: Polymerase chain reaction
PTS: Phosphotransferase system
SNP: Single nucleotide polymorphism
