# Supplementary figures and images for: Expanding the biodiversity of Oenococcus oeni through comparative genomics of apple cider and kombucha strains
Source: BMC Genomics. 2019 May 2;20:330. doi: 10.1186/s12864-019-5692-3 (PMC6498615; doi:10.1186/s12864-019-5692-3)

PSU-1

1

CRBO\_1381

1

UBOCC-A-315001

1

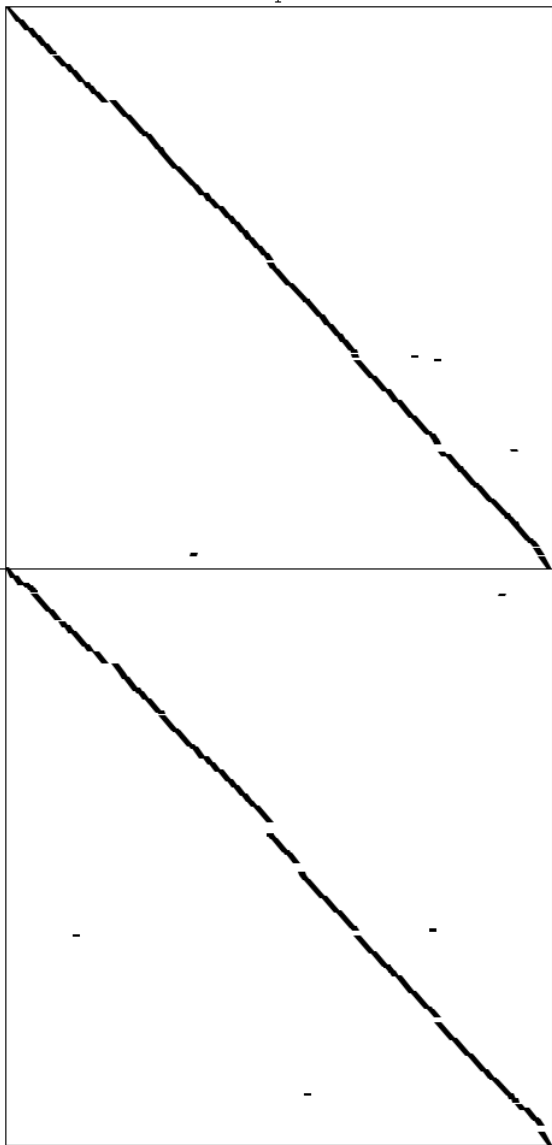

Supplement: Supplementary file 1 — Figure S1. Whole Genome Synteny Dotplot. Sequences of CRBO_1381 and UBOCC-A-315001 were compared against PSU-1 using SyMap. The algorithm finds pairwise genome alignment ‘anchors’ - represented by dots - and computes blocks of synteny (PDF 11 kb) [file 12864_2019_5692_MOESM1_ESM.pdf]

# Pangenome progression

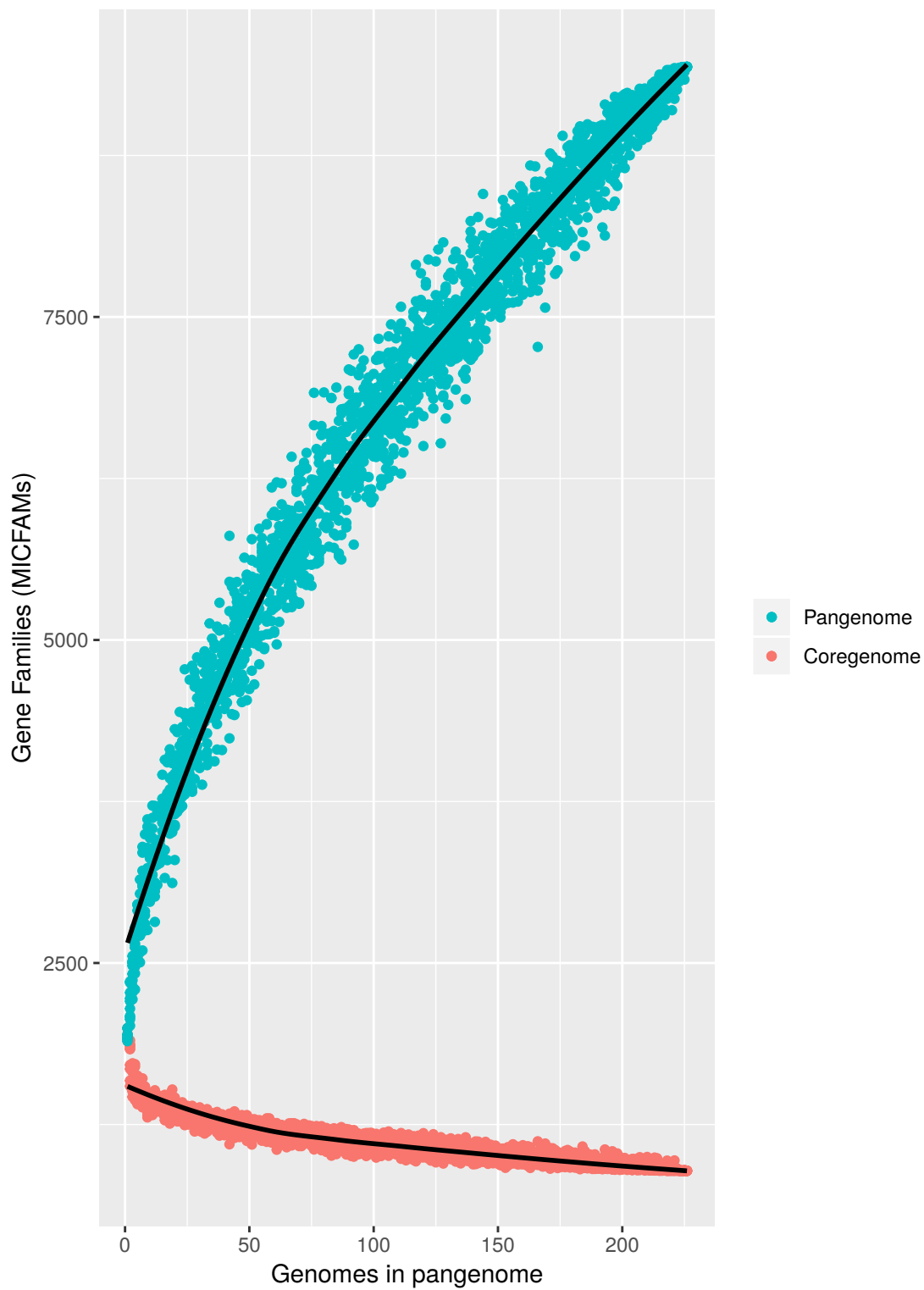

Supplement: Supplementary file 3 — Figure S3. Size of the pangenome at any given number of strains. At every step, 10 combinations of strains were randomly sampled within the total distribution. A locally weighting smoothing (loess) regression line was drawn for both sets (PDF 232 kb) [file 12864_2019_5692_MOESM3_ESM.pdf]

*O. oeni* UBOCC-A-315001

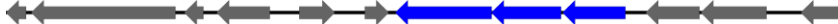

*O. kitaharae* DSM\_17330

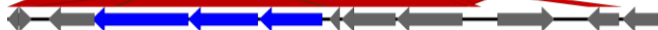

*C. botulinum* A Hall

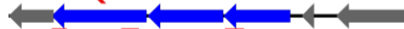

*S. pyogenes* HSC5

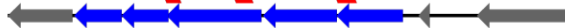

*sagF*

*sagE*

*sagD*

*sagC*

*sagB*

1 Kbp

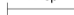

Supplement: Supplementary file 4 — Figure S4. Comparison of genomic regions overlapping a streptolysin operon. Pairwise BLAST hits shown in red (e < 0.001), darker color indicates better alignment. Blue: Streptolysin-associated genes. Grey: Genes outside syntenic operon. Related genes detected by synteny at minimum 26% protein identity (PDF 62 kb) [file 12864_2019_5692_MOESM4_ESM.pdf]

## Phylogroups

- A
- B
- C
- D

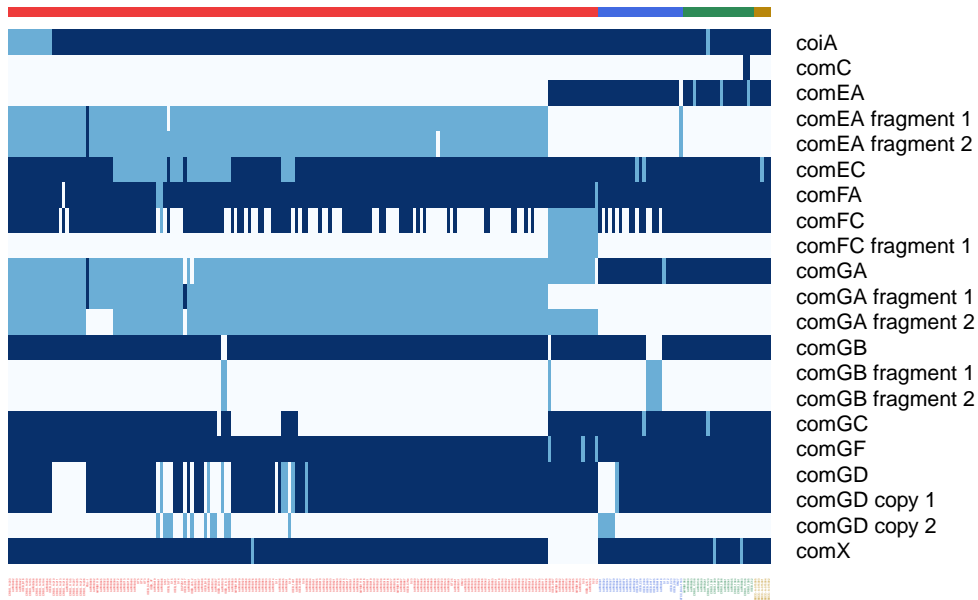

Supplement: Supplementary file 5 — Figure S5. Competence genes identified in the pangenome. Gene presence in blue, fragments in light blue (PDF 22 kb) [file 12864_2019_5692_MOESM5_ESM.pdf]

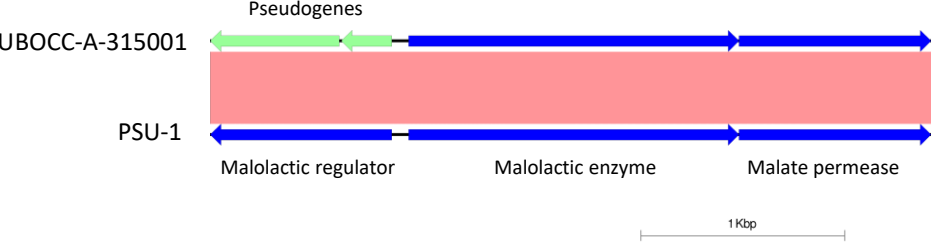

Supplement: Supplementary file 6 — Figure S6. Schematic representation of the stop mutation disrupting the malolactic transcriptional regulator in group D strains compared with PSU1 (PDF 29 kb) [file 12864_2019_5692_MOESM6_ESM.pdf]

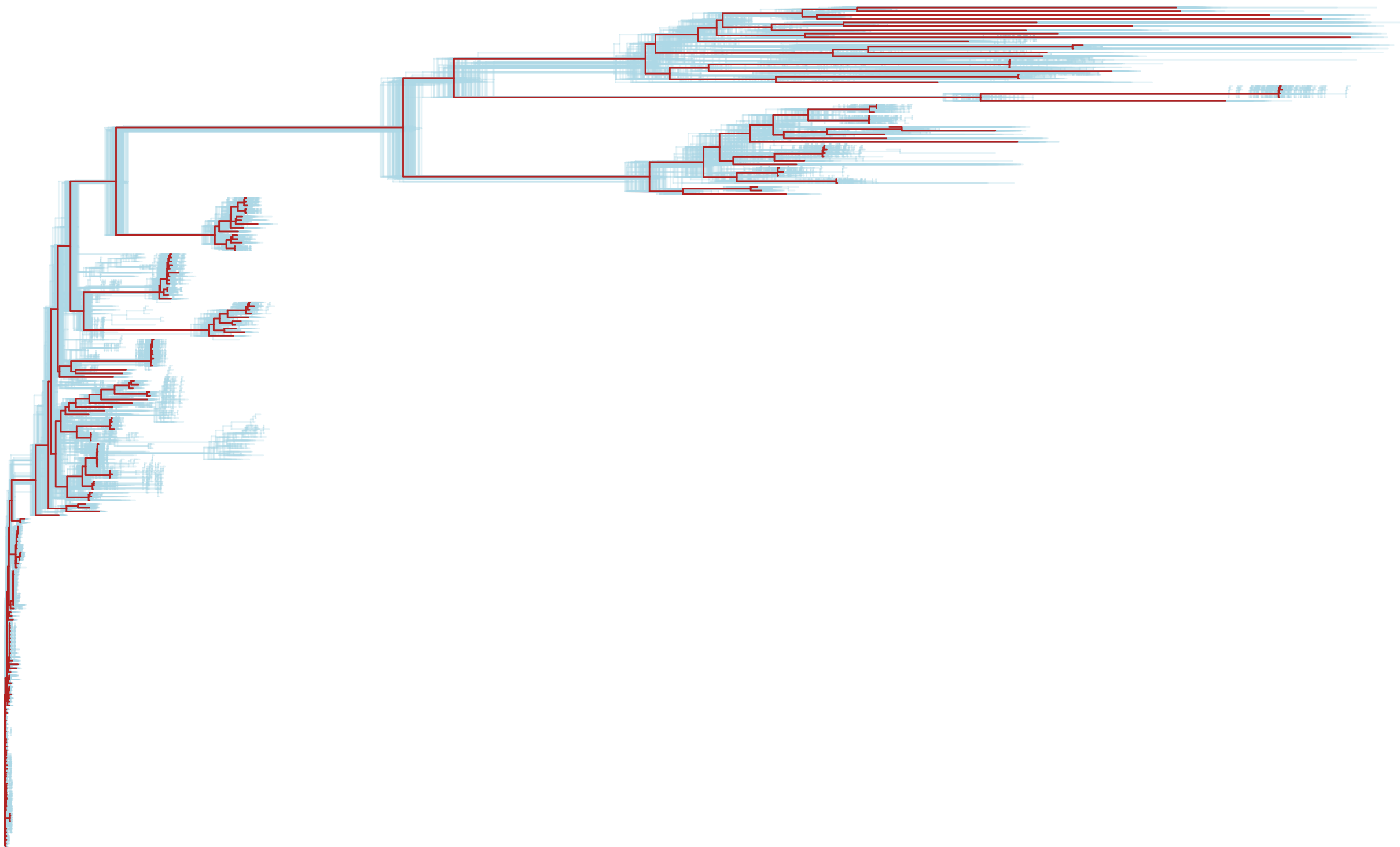

Supplement: Supplementary file 7 — Figure S7. Fortified Neighbor Joining phylogram. Calculated from core SNP data with Kimura 2-parameter distances, bootstrap n = 100 (PDF 717 kb) [file 12864_2019_5692_MOESM7_ESM.pdf]
