# Supplementary material for: Expanding the biodiversity of Oenococcus oeni through comparative genomics of apple cider and kombucha strains
Source: BMC Genomics. 2019 May 2;20:330. doi: 10.1186/s12864-019-5692-3 (PMC6498615; doi:10.1186/s12864-019-5692-3)
Supplement: Supplementary file 2 — Figure S2. Variable regions in groups C and D genomes. MicroScope RGP-finder was used to identify specific regions of (a) group C strain CRBO_1381 against the 5 group D strains and of (b) group D strain UBOCC-A-315001 compared to the 21 group C strains. Specific regions are shown in grey. Supporting algorithms are shown in blue and black (Interpolated Variable Order Motifs and Regions of Genomic Plasticity). tRNAs are in pink. (c) MaGe’s RGP-finder tool was employed to locate all variable regions, determine their size and the number of CDS they contain (PDF 466 kb) [file 12864_2019_5692_MOESM2_ESM.pdf]

a

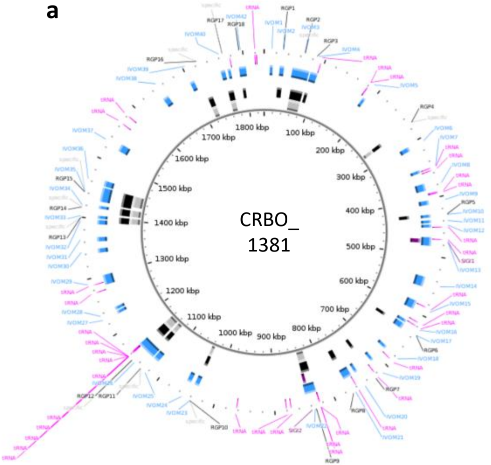

b

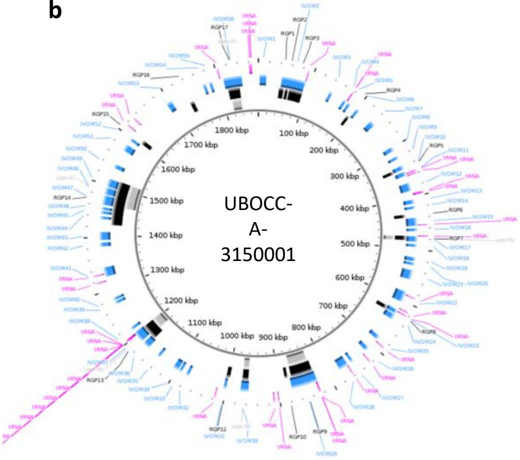

c

Strains	Tested against	Regions	Total length (bp)	Range of regions (bp)	CDS
CRBO_1381	5 D-strains	10	143,095	6,341-28,417	177
UBOCC-A-315001	21 C-strains	6	208,765	8,355-89,353	273
